# Microstructure, local electronic structure and optical behaviour of zinc ferrite thin films on glass substrate

**DOI:** 10.1098/rsos.181330

**Published:** 2018-10-17

**Authors:** Jitendra Pal Singh, Byeong-Hyeon Lee, Weon Cheol Lim, Cheol-Hwee Shim, Jihye Lee, Keun Hwa Chae

**Affiliations:** Advanced Analysis Center, Korea Institute of Science and Technology, Seoul 02792, South Korea

**Keywords:** thin films, zinc ferrite, local electronic structure, optical studies

## Abstract

Zinc ferrite thin films were deposited using a radio-frequency-sputtering method on glass substrates. As-deposited films were annealed at 200°C for 1, 3 and 5 h, respectively. X-ray diffraction studies revealed the amorphous nature of as-grown and annealed films. Thickness of as-deposited film is 96 nm as determined from Rutherford backscattering spectroscopy which remains almost invariant with annealing. Transmission electron microscopic investigations envisaged a low degree of crystalline order in as-deposited and annealed films. Thicknesses estimated from these measurements were almost 62 nm. Roughness values of these films were almost 1–2 nm as determined from atomic force microscopy. X-ray reflectivity measurements further support the results obtained from TEM and AFM. Near-edge X-ray absorption fine structure measurements envisaged 3+ and 2+ valence states of Fe and Zn ions in these films. UV–Vis spectra of these films were characterized by a sharp absorption in the UV region. All films exhibited almost the same value of optical band gap within experimental error, which is close to 2.86 eV.

## 1. Introduction

Apart from immense applications of crystalline thin films, amorphous thin films also provide ample opportunities for using them in tremendous applications. Metallic glass thin films (i.e. TiCuNi) are effective for biomedical applications due to average roughness of less than 0.2 nm, high wear resistance, high mechanical properties (hardness 6.9 GPa and reduced modulus 130 GPa) and surface free from Ni [1,2]. Amorphous metallic thin films (AMTFs) are of potential use for metal–insulator–metal tunnel diode applications due to their ultra-smooth surfaces, a consequence of their amorphous microstructure [3–5]. Amorphous thin films are important, but research activities in this direction are not significant. Thus, we focused our attention on investigating the behaviour of amorphous zinc ferrite thin films. The unit cell of zinc ferrite is formed by face-centred cubic arrangement of O^2−^ ions. Zn^2+^ and Fe^3+^ ions occupy tetrahedral (*A*-site) and octahedral (*B*-site) sites formed by 4 and 6 O^2−^ ions, respectively [6]. In zinc ferrite, properties are affected by the cation redistribution and a lot of reports are available establishing a relationship between the microstructure of these films and cation redistribution [7–9]. Thus, microstructural properties are more important in the case of amorphous films. This means there is a need to investigate the microstructural behaviour of these films as the properties are sharply dependent on microstructure [10–12]. Previous studies envisage modulation of the microstructure, varying thermal treatment and transformation of amorphous phase to crystalline with the increase of annealing temperature [13–15]; however, the influence of varying annealing time is hardly reported. In fact, annealing temperature promotes crystallization in thin films. Thus, an attempt has been made to get atomistic control over thin film by varying annealing time at moderate annealing temperature. It is well established that the nature of substrate plays an important role in determining the growth of films. The crystalline orientation of films depends on the orientation of the substrate [16–18]. Thus, a substrate which itself has an amorphous nature will be suitable for the present investigation. Hence, the present work is motivated to grow zinc ferrite thin film on amorphous substrate and to investigate the effect of annealing time on the microstructural behaviour.

In this context, techniques like high-resolution transmission electron microscopy (HRTEM) and atomic force microscopy (AFM) are the most suitable to investigate the microstructure of thin films. Furthermore, X-ray absorption fine structure measurements at near edge throw light on the local electronic structure of materials [19–21]. These measurements provide information on the metallic valence state, hybridization and metal–metal/oxygen interaction in the materials [19–23]. It is well established that zinc ferrite is important for its magnetic response [6,7,19–23]; however, the optical behaviour of this material is equally important from the fundamental and application point of view. Hence, optical studies of zinc ferrite nanoparticles and thin films were carried out using UV–Vis spectroscopy in order to strengthen the understanding of optical absorption in nano dimensions [24–26]. It is also shown that, depending upon the composition and thickness of the film, zinc ferrite has the potential to be used for photocatalytic application [27–29]. Thus, the importance of this material in photocatalytic applications motivated us to study the optical behaviour of deposited zinc ferrite thin films. Hence, the present work investigates the microstructure, local electronic structure and optical response of zinc ferrite thin films.

## 2. Experimental details

### 2.1. Film growth and annealing

ZnFe_2_O_4_ thin films were deposited on glass substrate using the radio-frequency (RF)-sputtering method at the base pressure better than 2.5 × 10^−6^ Torr [30]. A ZnFe_2_O_4_ target procured from Alfa Aesar was used for depositing films. RF power, substrate temperature and target to substrate distance were 100 W, room temperature and 5 cm, respectively. Oxygen pressure of 20 mTorr was maintained during deposition and depositions were carried out for 40 min. Deposited films were annealed at 200°C for 1, 3 and 5 h in air. Deposited and annealed films are coded as GZFNA, GZF21, GZF23 and GZF25 according to annealing temperature and time, respectively.

### 2.2. Film characterization

A crystalline phase of the target was determined using a D/MAX2500 (Rigaku, Japan) X-ray diffractometer with Cu K*α* (*λ* = 1.5418 Å) radiation. The semi-quantitative analysis (SQA) of the target was carried out using a ZSX Primus II (Rigaku) X-ray fluorescence spectrometer. X-ray diffraction (XRD) measurements were performed at one-dimensional (1D) XAS KIST-PAL beamline equipped with a bending magnet as the X-ray source by adjusting X-ray beam energy to 8049 keV. Rutherford backscattering (RBS) spectra of these films were obtained using an NEC Pelletron 6SDH-2 Accelerator. The depth resolution of the RBS spectrometer is 10 nm. RBS spectra of these films were simulated using RUMP software [31]. A Titan 80–300™ transmission electron microscope was used to determine thickness and crystalline quality of these films. The surface information was obtained using an XE-100 (Park Systems, Korea) AFM. X-ray reflectivity measurements were performed using ATX-G (Rigaku) X-ray diffractometer. XRR curves were simulated using X'Pert reflectivity.

The near-edge X-ray absorption fine structure (NEXAFS) measurements for these films were performed at the soft X-ray 10D XAS KIST beamline operating at 3.0 GeV with a maximum storage current of 400 mA. The NEXAFS spectra of these films were collected in the total electron yield (TEY) and total fluorescence yield (TFY) mode at room temperature in vacuum approximately 1.5 × 10^−8^ Torr [32]. NEXAFS spectra of all films along with reference oxides (FeO and Fe_2_O_3_) were recorded with step size of 0.1 eV [31,32]. Fe *K*-edge and Zn *K*-edge X-ray absorption near-edge absorption spectra (XANES) measurements were performed on 1D XRD-KIST beamline based on bending magnet. Schematics of XANES measurements are depicted in electronic supplementary material, figure S1. The detailed description is given elsewhere [33]. The program Athena was used to identify the beginning of the absorption edge (*E*_0_), fit pre- and post-edge backgrounds, and hence to obtain the normalized spectra [34,35]. Optical behaviour of these films was determined using UV–visible spectra in transmittance using a Varian Cary-100 UV–visible spectrophotometer.

## 3. Results and discussion

### 3.1. Phase and thickness analysis

**Figure 1.**
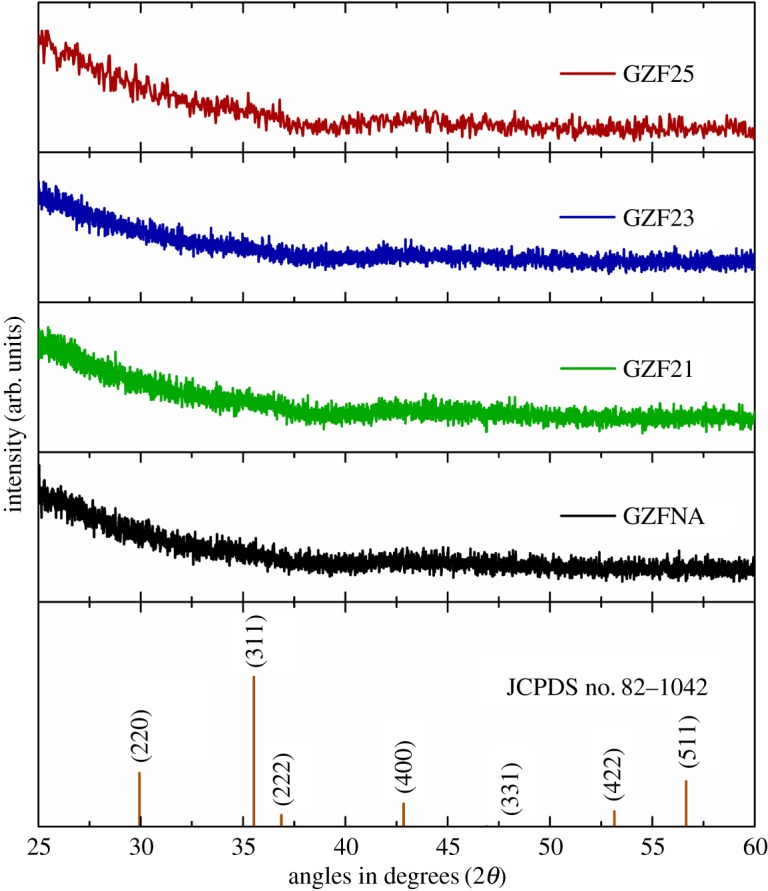
X-ray diffraction patterns of zinc ferrite thin films.

**Figure 2.**
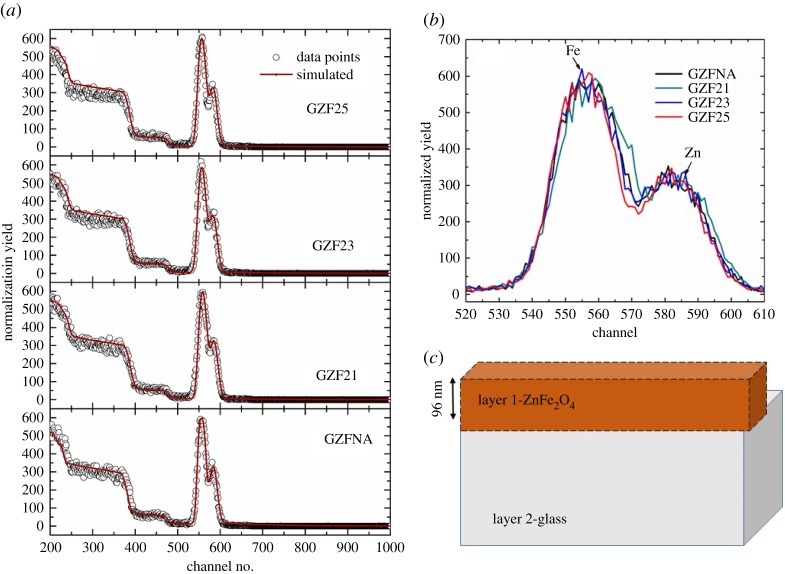
(*a*) Simulated RBS spectra of GZFNA, GZF21, GZF23 and GZF25 thin films. (*b*) Inset shows RBS spectra of GZFNA, GZF21, GZF23 and GZF25 to highlight Zn and Fe regions. (*c*) Model used for simulation of RBS spectra of various thin films.

XRD patterns of GZFNA, GZF21, GZF23 and GZF25 are shown in figure 1. As-deposited GZFNA film is amorphous in nature. It is well established that post deposition annealing is essential to crystallize ferrite thin films [21,23,27]; however, XRD patterns of GZF21, GZF23 and GZF25 envisage that films are amorphous even after annealing. Before discussing the nature of deposited films, the phase and stoichiometry of the procured target was also investigated. It is observed that the crystalline phase of the target is cubic spinel (electronic supplementary material, figure S2). SQA measurements reflect that the Zn : Fe : O ratio is 1 : 2 : 4 which reveals that the target is perfectly stoichiometric (electronic supplementary material, table S1). Thus, amorphous ZnFe_2_O_4_ thin films are formed from the polycrystalline ZnFe_2_O_4_ target under these growth parameters. This indicates that annealing at 200°C is not sufficient to promote crystallization in zinc ferrite thin films [36]. Results obtained from RBS studies are shown in figure 2. Figure 2*a* shows simulated RBS spectra of GZFNA, GZF21, GZF23 and GZF25. Figure 2*b* shows the measured RBS spectra in the specific region to highlight the spectral feature Zn and Fe elements. In figure 2*c*, the model used for simulation is collated. It is clear that widths of spectral features corresponding to Zn and Fe elements remain almost invariant for these films. Thicknesses of these films are close to 96 nm as estimated from simulation of these spectra. This indicates that there is no significant change occurring with annealing.

**Figure 3.**
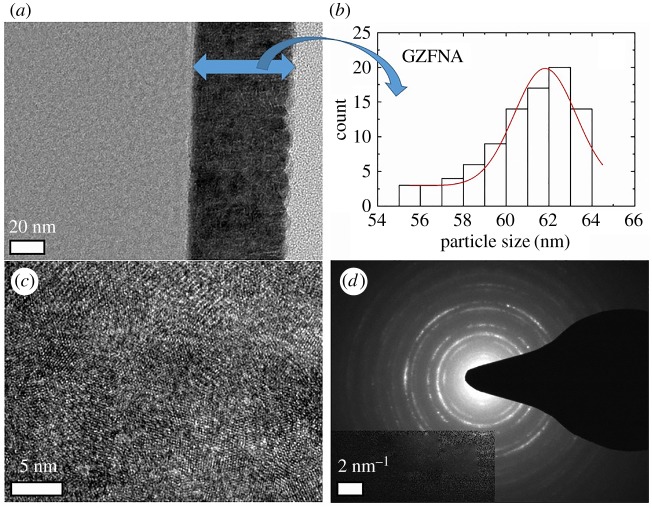
(*a*) TEM image, (*b*) thickness distribution, (*c*) lattice structure and (*d*) selected area diffraction pattern of GZFNA.

**Figure 4.**
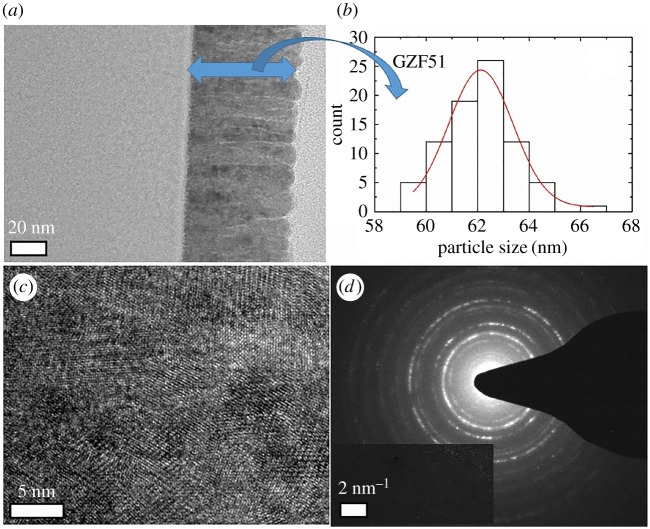
(*a*) TEM image, (*b*) thickness distribution, (*c*) lattice structure and (*d*) selected area diffraction pattern of GZF25.

**Figure 5.**
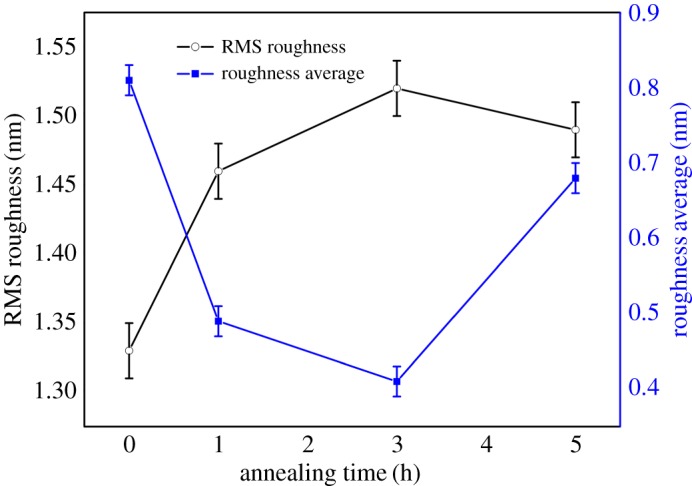
Root mean squares (RMS) and average roughness of thin films with annealing time determined from atomic force microscopy.

**Figure 6.**
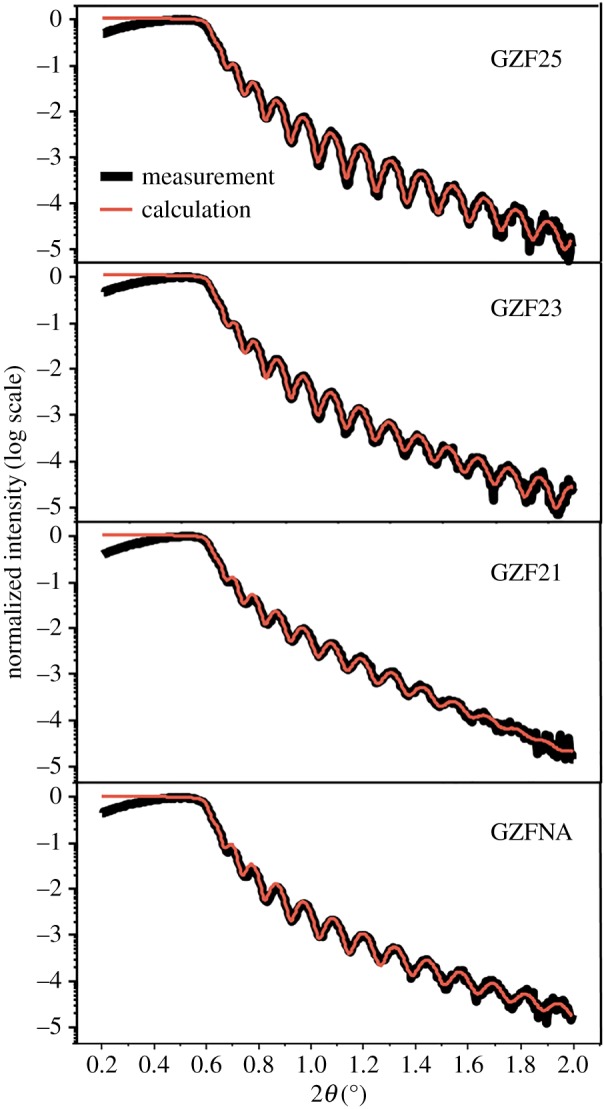
Experimental and calculated X-ray reflectivity curves of GZFNA, GZF21, GZF23 and GZF25 thin film.

Both the crystalline order and thickness are further investigated using TEM to corroborate results obtained from XRD and RBS. Figure 3 shows results obtained from TEM investigation for as-deposited GZFNA film. Figure 3*a* shows formation of a continuous layer during deposition. Thickness of this layer is 62 ± 2 nm as determined from the Gaussian fitting of various measurement performed across the film (figure 3*b*). Figure 3*c* shows a lattice image of this film. Lattice structures of these film do not reveal the presence of long-range ordering; however, a selected area diffraction (SAD) pattern of GZFNA film exhibits rings which envisage poor crystallinity of as-deposited film [37,38]. To depict the change associated with annealing a TEM study of this film was also performed for GZF25 film as shown in figure 4. Figure 4*a* reveals the presence of a continuous but slightly disordered layer. The thickness of this layer is almost identical to that of as-deposited counterpart. The thickness of this layer is 63 ± 2 nm (figure 4*b*). A lattice image of this layer, shown in figure 4*c*, exhibits slightly improved crystallinity. However, the presence of a diffused ring in the SAD pattern for GZF25 film again reveals the onset of a poor degree of crystallinity [37,38]. The surface quality of these films is determined from the AFM shown in electronic supplementary material, figure S3. These films also exhibit the absence of granular nature, making them continuous films. Estimated values of roughness are close to 1 for these films (figure 5). The thickness and surface roughness were further measured using XRR measurements. Figure 6 shows the experimental and simulated curves for these films. These curves were simulated by considering three layers, namely (i) surface layer, (ii) film layer, and (iii) interface layer. The results obtained are collated in table 1. It is clear from these results that the thickness of films is comparable to that estimated from TEM. However, the values of thicknesses are low compared with those determined from RBS. This effect may also be due to the ability of the RBS technique to detect diffused ions in the substrate [31].

**Table 1.** Thickness (nm), density (g cm^−3^) and roughness (nm) for GZFNA, GZF21, GZF23 and GZF25 thin films.

layers	material	thickness (nm)	density (g cm^−3^)	roughness (nm)
GZFNA				
3	ZnFe_2_O_4_	0.80	2.25	0.94
2	ZnFe_2_O_4_	63.06	4.82	3.25
1	ZnFe_2_O_4_	2.90	2.92	1.99
sub.	glass	n.a.	2.65	1.00
GZF21				
3	ZnFe_2_O_4_	1.20	3.90	1.25
2	ZnFe_2_O_4_	63.50	4.91	3.20
1	ZnFe_2_O_4_	4.40	2.94	2.00
sub.	glass	n.a.	2.65	1.00
GZF23				
3	ZnFe_2_O_4_	0.90	2.77	1.20
2	ZnFe_2_O_4_	64.19	4.95	3.70
1	ZnFe_2_O_4_	4.68	2.59	2.47
sub.	glass	n.a.	2.65	1.00
GZF25				
3	ZnFe_2_O_4_	1.77	2.38	1.06
2	ZnFe_2_O_4_	63.40	4.95	2.97
1	ZnFe_2_O_4_	3.19	2.43	2.00
sub.	glass	n.a.	2.65	1.00

### 3.2. Local electronic structure investigations

**Figure 7.**
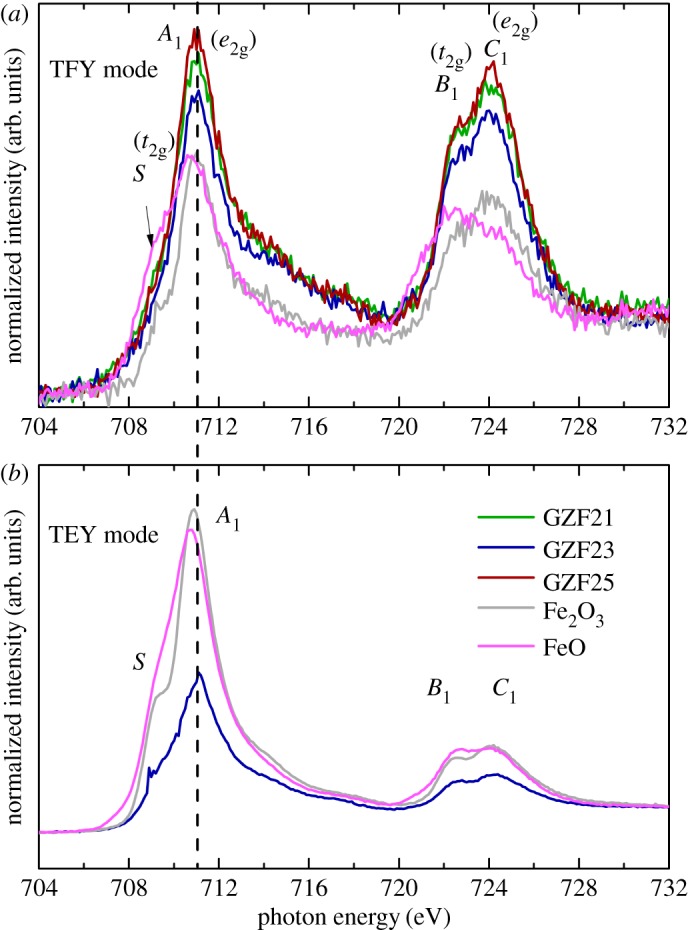
(*a*) Fe *L*-edge spectra of GZF21, GZF23 and GZF25 thin films in TFY mode along with Fe_2_O_3_ and FeO. (*b*) TEY mode NEXAFS spectra for GZF23 thin film along with Fe_2_O_3_ and FeO.

Figure 7 shows the Fe *L*-edge spectra of GZF21, GZF23 and GZF25 along with FeO and Fe_2_O_3_. TFY Fe *L*-edge spectra of these films are shown in figure 7*a*, along with the reference. The spectral features appearing in these spectra are analogues to the oxides of iron, which indicate the presence of Fe-oxidation in these films. Spectra of these films exhibit a shoulder S along with spectral features *A*_1_, *B*_1_ and *C*_1_. These spectral features are associated with *e*_g_ (*L*_3_) and *t*_2 g_ (*L*_2_) and *e*_g_ (*L*_2_) symmetry states. Generally, Fe-based oxides also exhibit spectral corresponding to *t*_2 g_ (*L*_3_) symmetry states [19,39,40]. In the present case, this state is evidenced by the presence of shoulder S in the spectra of amorphous thin films. It is clear that the position of *A*_1_ spectral features of these films are almost the same as that of Fe_2_O_3_; however, the position of the *A*_1_ spectral feature of FeO is preceded by 0.4 eV to that of these films (electronic supplementary material, figure S4). Thus, it is suggested that Fe valence state is 3+ in these films [13]. It is well known that the properties of films are affected by surface, hence information on Fe at the surface of the film was studied by measuring NEXAFS spectra in TEY mode. Figure 7*b* shows Fe *L*-edge TEY NEXAFS spectra of representative GZF23 films along with reference oxides. The TEY NEXAFS spectrum of this film exhibits a shoulder-like feature S along with spectral features *A*_1_, *B*_1_ and *C*_1_. These spectral features are also present in the TFY NEXAFS spectra of these films, which indicates that the surface and bulk of these films exhibit almost same electronic structure. The *A*_1_ spectral feature of this film also appears to almost coincide with that of Fe_2_O_3_. This envisages that Fe resides in 3+ valence state at the surface of the film. Splitting of the *A*_1_ spectral feature gives important information about the valence state and crystalline field. The presence of Fe in octahedral field gives rise to dominant splitting; however, splitting remains almost negligible in the presence of tetrahedral field [39–41]. Thus, shoulder *S*, instead of being a prominent peak, is associated with the migration of Fe to *A*-site of the spinel structure.

**Figure 8.**
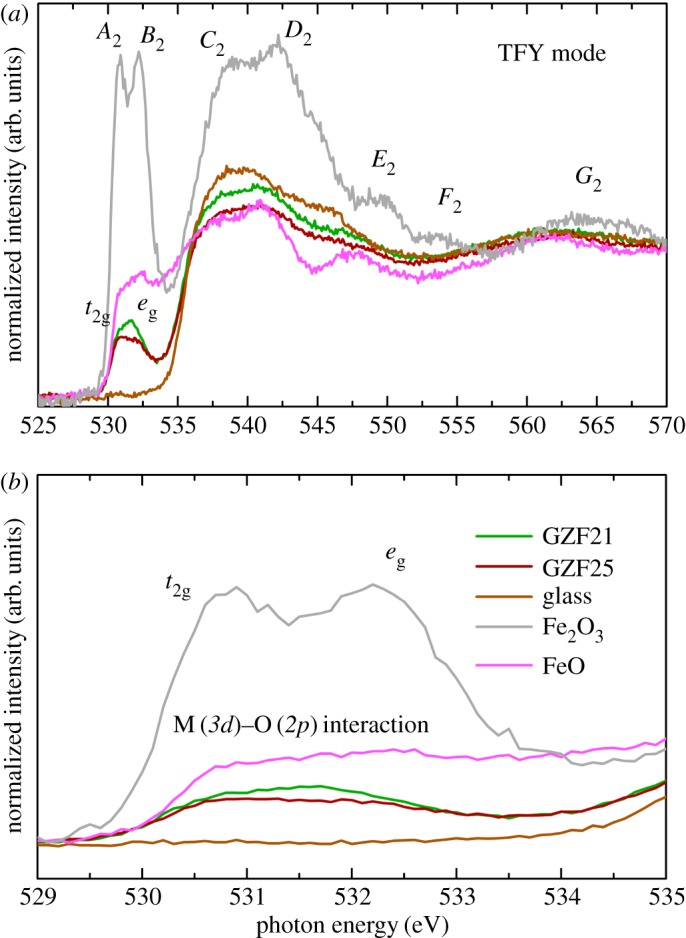
(*a*) O *K*-edge spectra of different thin films in TFY mode along with Fe_2_O_3_ and FeO. (*b*) Pre-peak region shows the absence of splitting to *t*_2 g_ and *e*_g_ states.

**Figure 9.**
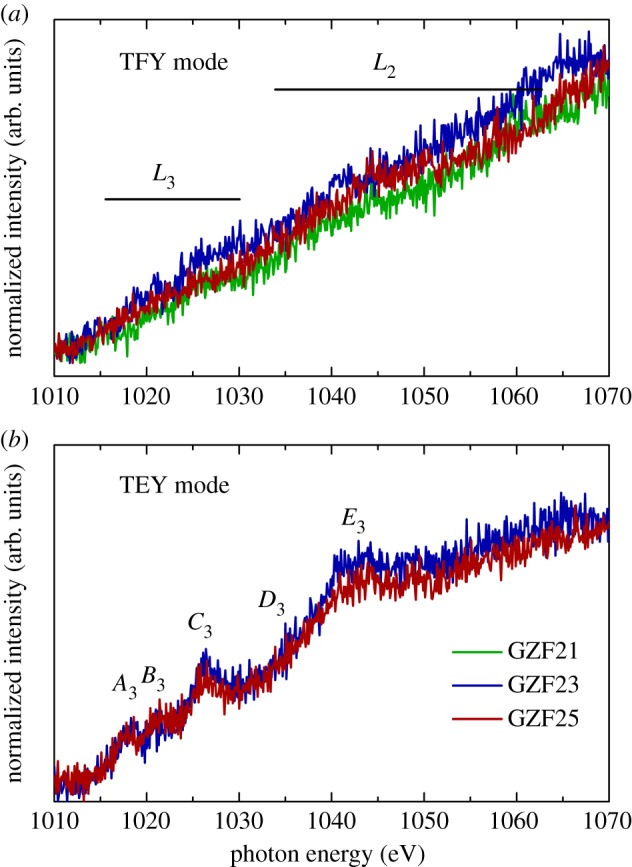
(*a*) Zn *L*-edge spectra of different thin films in (*a*) TFY and (*b*) TEY modes.

To understand the metal–oxygen interaction, TFY O *K*-edge spectra of representative films are measured and shown in figure 8*a* along with the spectrum of glass, FeO and Fe_2_O_3_. These spectra exhibit spectral features *A*_2_, *B*_2_, *C*_2_, *D*_2_, *E*_2_, *F*_2_ and *G*_2_ that are generally present in Fe-based oxides. Pre-edge spectral features *A*_2_ and *B*_2_ (figure 8*b*), which are absent in the spectrum of glass, give important information about metal–oxygen interaction. In the case of zinc ferrite, the pre-edge region has contributions from Fe(3*d*)–O(2*p*) and Zn(3*d*)–O(2*p*) symmetry states indicating the interaction of Fe and Zn with the oxygen [13,41,42]. Thus, the presence of pre-edge spectral features in the present case reflects interaction between metal and oxygen. Figure 9 shows Zn *L*-edge spectra of GZF21, GZF23 and GZF25 in TFY and TEY mode. In TFY mode NEXAFS spectra of these films, spectral features are almost absent (figure 9*a*). TEY Zn *L*-edge spectra of representative GZF23 and GZF25 thin films contain spectral features *A*_3_, *B*_3_, *C*_3_, *D*_3_ and *E*_3_. These spectral features are also reported for zinc ferrite nanoparticles of different sizes in our previous work [13,40]. The presence of these spectral features is associated with 2+ valence state of Zn ions in ZnFe_2_O_4_. Thus, the surfaces of these films contain Zn^2+^ ions, and the absence of these spectral features is due to low resolution of the beam in TFY mode.

**Figure 10.**
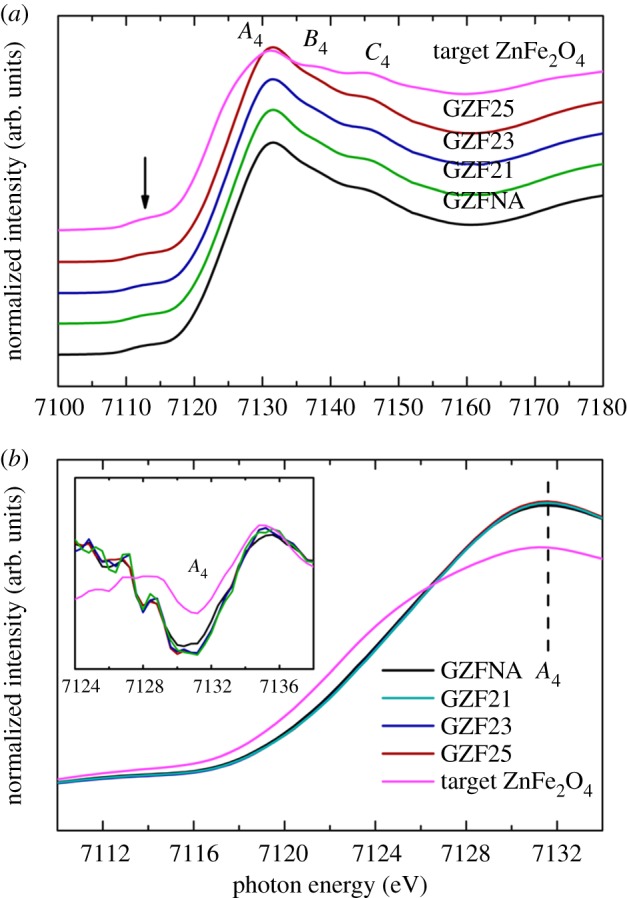
(*a*) Fe *K*-edge spectra at near edge for GZFNA, GZF21, GZF23 and GZF25 with target ZnFe_2_O_4_. (*b*) Spectral feature *A*_4_ of these spectra. Inset in (*b*) shows the second derivative at main edge.

**Figure 11.**
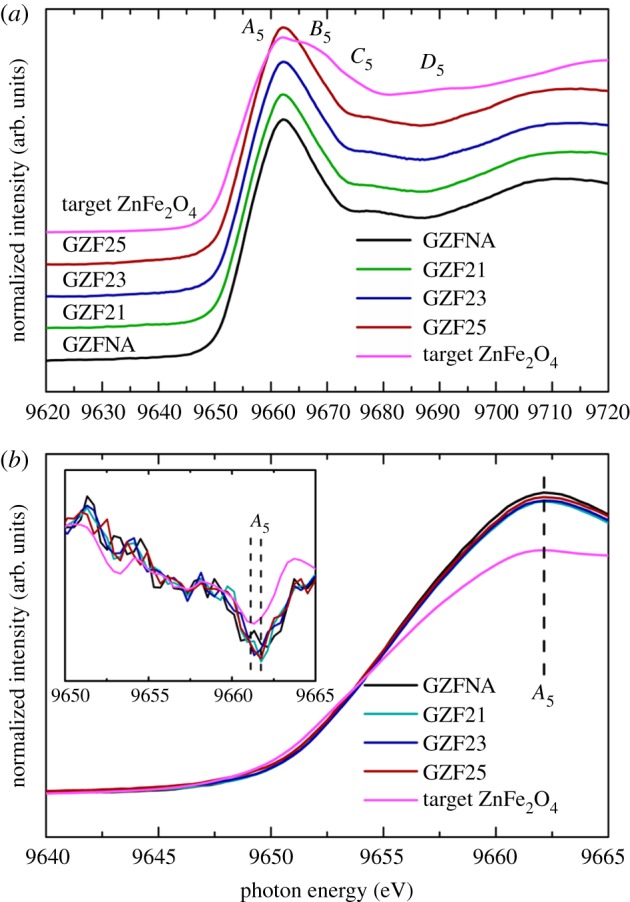
(*a*) Zn *K*-edge spectra at near edge for GZFNA, GZF21, GZF23 and GZF25 with target ZnFe_2_O_4_. (*b*) Spectral feature *A*_5_ of these spectra. Inset in (*b*) shows the second derivative at main edge.

Fe *K*-edge spectra of these films are shown in figure 10. In the present case, spectral features *A*_4_, *B*_4_ and *C*_4_ appear in the spectra of GZFNA, GZF21, GZF23, GZF25 and target ZnFe_2_O_4_ (figure 10*a*). These spectral features also appear in the Fe *K*-edge spectra of zinc ferrite nanoparticles [20–22,40]. In the spectra, the behaviour of spectral feature *A*_4_ is different to that of bulk ZnFe_2_O_4_ (figure 10*b*). The position of this spectral feature is slightly different to that of target (figure 10*b*, inset). Zn *K-*edge spectra measured for these films exhibit spectral features *A*_5_, *B*_5_ and *C*_5_ (figure 11). These spectral features also appear in the spectra of target ZnFe_2_O_4_ and are in agreement with previous studies [20–22,41]. Spectral feature *A*_5_ in these films is sharper compared with the spectra of the target. The *A*_5_ spectral feature of these films exhibits significant with respect to target ZnFe_2_O_4_.

### 3.3. Optical behaviour

**Figure 12.**
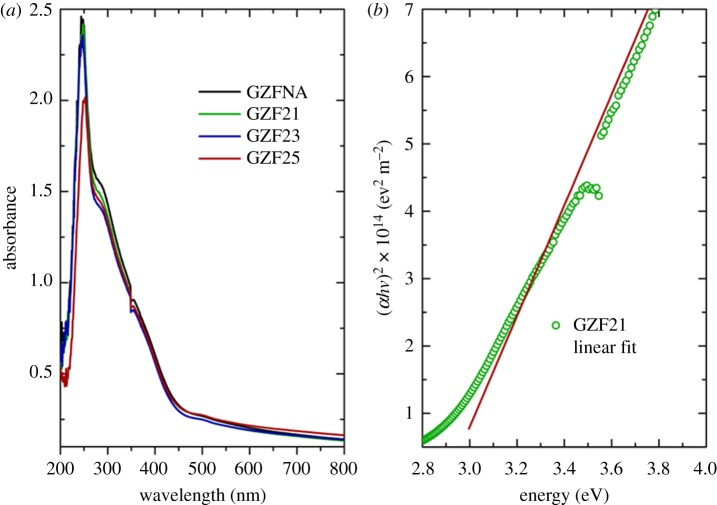
(*a*) UV–Vis spectra of ZnFe_2_O_4_ thin films and (*b*) (*αhv*)^2^ versus *hυ* for estimation of direct optical band gap of representative GZF21 thin film.

Thus, these thin films exhibit a local electronic structure which is different from bulk ZnFe_2_O_4_. This effect is associated with a low degree of crystallization. To investigate optical behaviour associated with low crystallinity, UV–Vis spectra of these films were measured. Figure 12*a* shows the UV–Vis spectra measured for these films. All films exhibit strong absorption in UV region. Similar behaviour of optical absorption is also reported for zinc ferrite thin films [25–27,29,30] and nanoparticles [24,28,43]. The optical band gaps (*E*_g_) of these films were estimated from the Tauc's relation as follows:

$$\alpha hv=A{\left(hv-E_{g}\right)}^{m},$$

where *A* is a constant, *hν* is the energy of incident radiation, *m* is an index showing the nature of transition, *m* = 1/2 for allowed direct and 3/2 for forbidden direct transitions. *α* is known as absorption coefficient and estimated using the following relation:

$$\alpha =\frac{2.303\mathrm{Abs}}{t},$$

where *t* is the thickness of film and Abs is the absorption of film. Figure 12*b* shows (*αhv*)^2^ versus *hv* for these films. Extrapolation of these curves to the *X*-axis gives a value of direct optical band gap [25,44]. Values of optical band gap are 2.89 ± 0.02, 2.88 ± 0.02, 2.86 ± 0.02 and 2.86 ± 0.02 eV for GZFNA, GZF21, GZF23 and GZF25 thin films, respectively. A direct band gap of 2.70 eV was also reported by Wu *et al*. [45] in crystalline zinc ferrite thin films.

## 4. Conclusion

The zinc ferrite thin films with a low degree of crystallization are grown using the RF- sputtering method. Annealing at 200°C for different annealing durations does not result in crystalline phase. RBS spectroscopy and high-resolution transmission electron microscopy reveal that thickness remains almost the same for several annealing durations, which is supported by X-ray reflectivity. Atomic force microscopy measurements show modification of surface roughness with annealing. The valence state and nature of hybridization are not affected by the annealing duration. These films exhibit optical band gap of approximately 2.86 eV, which remains almost constant within experimental error.

## Supplementary Material

Measurement procedure for obtaining XANES spectra

## Supplementary Material

XRD pattern of target ZnFe2O4

## Supplementary Material

Atomic force microscopic images for thin films

## Supplementary Material

Energy shift of spectral feature A1 of Fe L-edge TFY NEXAFS spectra of films along-with reference oxides.

## Supplementary Material

Semi-quantitative analysis for target ZnFe2O4

## Data accessibility

All supporting information like tables and figures are included in the electronic supplementary material. The content of electronic supplementary file is as follows: electronic supplementary material, table S1 shows the results obtained from semi-quantitative analysis for target ZnFe_2_O_4_. Measurement procedure for obtaining XANES spectra is shown in electronic supplementary material, figure S1. XRD pattern of target ZnFe_2_O_4_ is depicted in electronic supplementary material, figure S2. Atomic force microscopic images are shown in electronic supplementary material, figure S3. Electronic supplementary material, figure S4 reflects energy shift of spectral feature *A*_1_ of Fe *L*-edge TFY NEXAFS spectra of films along with reference oxides.
