# Supplementary figures and images for: Microstructure, local electronic structure and optical behaviour of zinc ferrite thin films on glass substrate
Source: R Soc Open Sci. 2018 Oct 17;5(10):181330. doi: 10.1098/rsos.181330 (PMC6227928; doi:10.1098/rsos.181330)

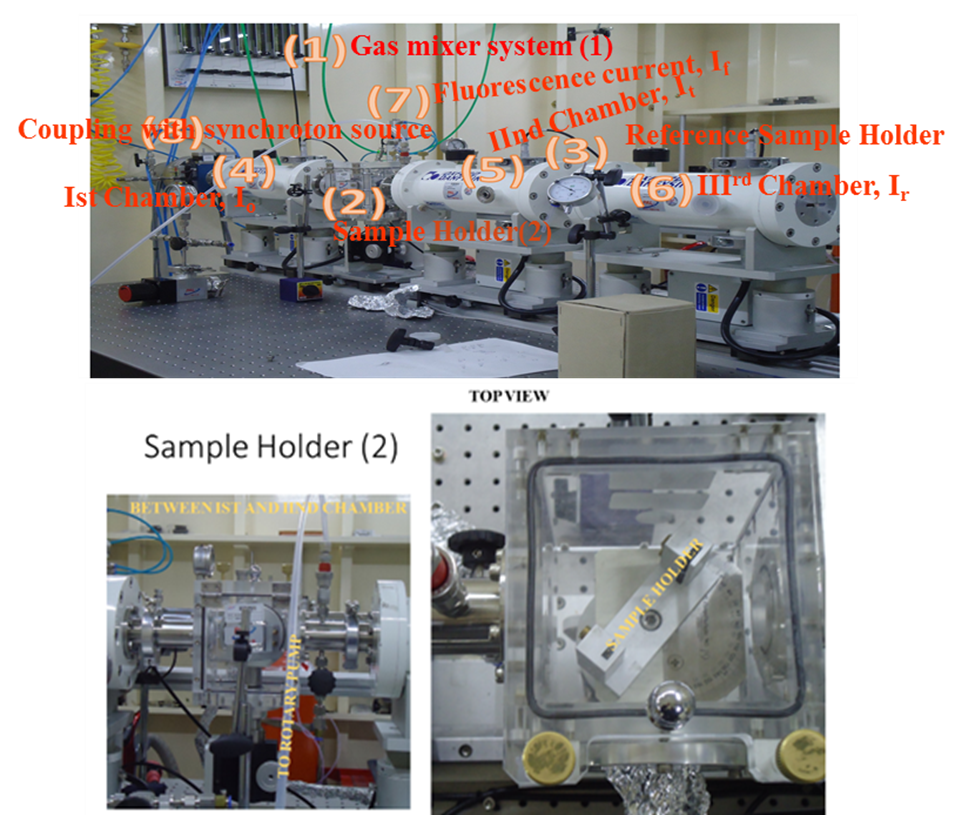

Supplement: Measurement procedure for obtaining XANES spectra [file rsos181330supp1.tif]

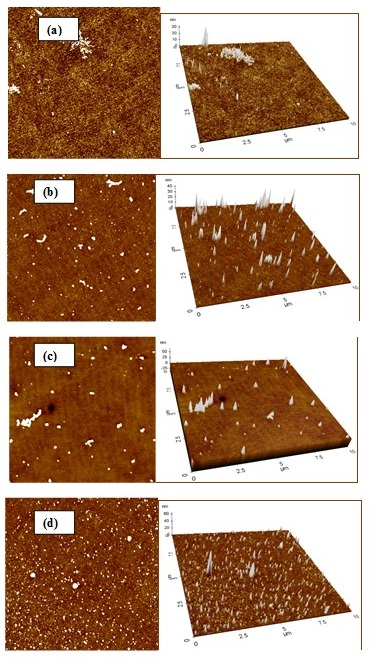

Supplement: Atomic force microscopic images for thin films [file rsos181330supp3.tif]

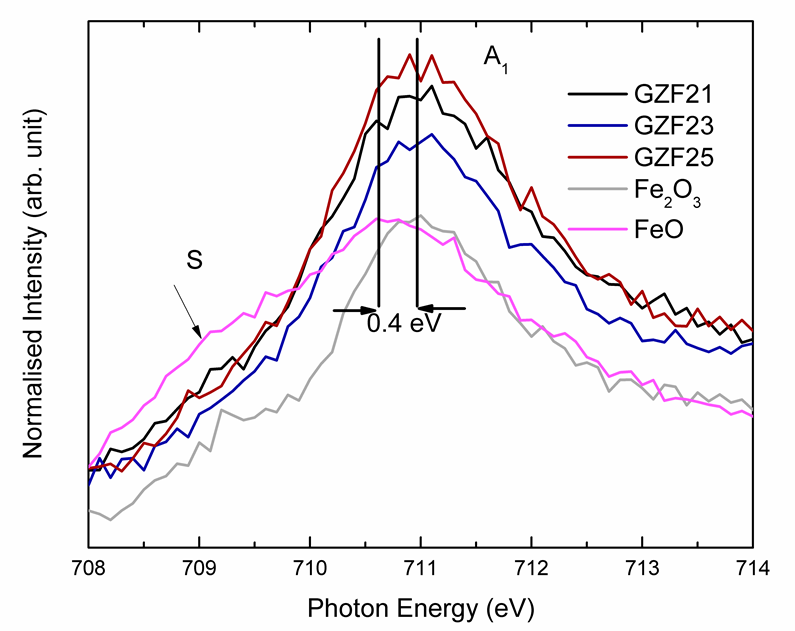

Supplement: Energy shift of spectral feature A1 of Fe L-edge TFY NEXAFS spectra of films along-with reference oxides. [file rsos181330supp4.tif]
