# Supplementary material for: Microstructure, local electronic structure and optical behaviour of zinc ferrite thin films on glass substrate
Source: R Soc Open Sci. 2018 Oct 17;5(10):181330. doi: 10.1098/rsos.181330 (PMC6227928; doi:10.1098/rsos.181330)
Supplement: Semi-quantitative analysis for target ZnFe2O4 [file rsos181330supp5.pdf]

SQX Calculation Result

Sample : Dr.채근화-타겟
Application : 5B~92U(Fil)10mm
Sample type : Metal & Alloy
Sample film corr. :
File : Dr.채근화-타겟
Date analyzed : 2018- 3-12 15:43
Balance :
Matching library :
Impurity corr. :

No.	Component	Result	Unit	Det. limit	El. line	Intensity	w/o normal
1	O	24.4	mass%	0.37111	O -KA	1.1919	24.9435
2	Al	0.0115	mass%	0.00408	Al-KA	0.0131	0.0118
3	Si	0.0291	mass%	0.00418	Si-KA	0.0349	0.0298
4	S	0.0307	mass%	0.00220	S -KA	0.0984	0.0315
5	Ca	0.0107	mass%	0.00459	Ca-KA	0.0412	0.0109
6	Mn	0.286	mass%	0.01145	Mn-KA	1.0142	0.2931
7	Fe	48.4	mass%	0.02314	Fe-KA	224.0285	49.5887
8	Zn	26.9	mass%	0.01638	Zn-KA	113.8263	27.5229
